# Underestimation and Overestimation of Hand and Arm Length Coexist in Children

**DOI:** 10.1111/desc.70035

**Published:** 2025-06-03

**Authors:** Lucilla Cardinali, Cristina Becchio, Lara Coelho, Monica Gori

**Affiliations:** ^1^ Department of Cognition, Motion and Neuroscience Istituto Italiano di Tecnologia Genoa Italy; ^2^ Department of Neurology University Medical Center Hamburg‐Eppendorf Hamburg Germany; ^3^ U‐VIP (Unit for Visually Impaired People) Istituto Italiano di Tecnologia Genoa Italy

**Keywords:** body representation, development, functional task, structural task

## Abstract

The present study assessed the structural and functional representation of the upper limb in a large cohort (*N* = 84) of typically developing children aged 6 to 10. The first task aimed at obtaining a structural measure of the representation of the arm, specifically the two segments that compose it: the forearm and the hand. Participants were asked to localize three landmarks (elbow, wrist, and tip of the middle finger) while blindfolded and upon tactile stimulation of the three landmarks. The second task required a functional estimation of the represented length of the arm. Participants judged whether their arm fully outstretched would be long enough to touch an object presented at seven different distances without being allowed to perform the movement. The two tasks revealed opposite patterns of (mis‐)representation. At the structural level, the hand length was underestimated, while the forearm representation matched the actual size. This resulted in an underestimation of total arm length in the structural task. At the functional level, total arm length was overestimated across all age groups. Moreover, there was no relationship between estimates on the structural and functional tasks. These results support the coexistence of multiple, independent body representations in children.

## 1 Introduction

The representation of one's body plays a fundamental role in shaping our lives across the lifespan. Specifically, body representations can influence higher cognitive functions and are fundamental for guiding our actions (Dijkerman and Lenggenhager 2018). Since the body is the main instrument for interacting with the world, accurate knowledge of its state is fundamental for effective planning and control. Imagine trying to reach or grasp an object with a distorted representation of one's arm or hand. Yet, distortions of body representations are not the exception but the rule (Bassolino and Becchio 2022; Coelho and Gonzalez 2024; Longo 2022). For example, previous research using a landmark task has reported a consistent underestimation of perceived finger length in adult populations accompanied by a significant increase in hand width (Caggiano et al. 2020; Coelho et al. 2023; Longo and Haggard 2010, 2012a; Longo et al. 2015; Tamè et al. 2017). In this task, participants are asked to localize various landmarks on their fingers. This task has consistently produced systematic distortions in hand representation. However, these distortions are reduced/eliminated in other tasks (Longo and Haggard 2010, 2012b). Specifically, hand size estimates in a body image task tend to be accurate. For example, in a template matching task, participants select the image that most closely resembles their own hand from an array of distorted photographs—and typically do so accurately. Nonetheless, Longo and Haggard (2012b) found that underestimation of finger length can extend beyond purely structural tasks. In that study, participants performed both the structural and template matching tasks, as well as a metric task in which they judged whether their finger was shorter or longer than a visual line. Interestingly, while structural representations were highly distorted and template matching remained accurate, metric representations retained some degree of distortion—though more accurate than the structural estimates. This supports the proposal of multiple body representations in the human brain (Anema et al. 2009; Gallagher and Cole 1995; Head and Holmes 1911; Paillard 1999) and further suggests that most representations are characterized by various, not always consistent, degrees of distortions. In fact, Longo and Haggard (2012b) argued that their results were evidence of two distinct body representations that can interact/combine to different extents depending on the task.

**Summary**

- During development structural arm length representation is underestimated, while the functional arm length representation is overestimated.
- Underestimation of structural arm length is driven by an underestimation of hand length, as forearm length is accurate.
- Structural hand length is underestimated, supporting that underestimation of hand length is a characteristic of human body representation.
- The opposite pattern of results between structural and functional arm representation suggests the existence of multiple independent representations of the body.

During childhood, changes in body size are considerable. Body representations are also being developed during this period based on both internal representations of the body as well as sensory feedback (e.g., vision and proprioception) through interactions with the environment (Von Hofsten 2004). Simultaneously, children are developing motor planning, spatial awareness, and action‐based decision‐making skills (Krajenbrink et al. 2020; Shi and Feng 2022). Importantly, as they grow, children refine their ability to predict and control their movements, suggesting an improved representation of the body (Campos et al. 2000). However, as we know that some body representations in adults remain distorted, what do body representations during childhood look like? In this article, we address how distorted body representations support functional behavior from a developmental perspective. Specifically, we aim to understand how developmental changes impact the structural representation of upper limb length (assessed using the landmark task), its potential for action (assessed using a reach estimation task), and their relationship. Body representation during development is an interesting topic because, unlike during adulthood, children have more constraints on judging their body size based on memory. In other words, because children undergo periods of rapid growth, their memory of their body size may not be as reliable. Therefore, if distortions of body representations exist, this may have a greater impact on action. To date no study has investigated both the structural and functional representation of upper limbs in the same group of children.

It has been shown that while adults consistently underestimate hand length, arm length is not always underestimated (Bassolino et al. 2015; Cardinali et al. 2011; Galigani et al. 2020). Moreover, previous research has established that in adult populations, the perceived size of the hand/arm influences perceived action capabilities. Specifically, right‐handed individuals estimated that they could reach farther with their right hand, grasp larger objects, and estimated their right arms/hands as being bigger (Linkenauger et al. 2009). This finding demonstrates a relationship between structural and functional body representations in adulthood. While hand and arm representation has been well studied in adults, there is less information about how these representations develop across childhood. There is, however, some evidence that children have different body representations than adults (Cardinali et al. 2019; Coelho and Gonzalez 2022; Cowie et al. 2022). For example, in a previous study assessing perceived hand and arm length, it was found that underestimation of hand length increases significantly from age 6–10, at which point it becomes an adult‐level of underestimation (Cardinali et al. 2019). In that study, the children were presented with different sized 3D‐printed hands and were asked to judge if each hand was bigger or smaller than their own hand. The authors argued that the underestimation of hand size indicated that body representations update slower than physical growth. Conversely, in tasks requiring action simulation, Gabbard and colleagues found the opposite pattern, specifically a significant overestimation of upper limb length (Gabbard and Ammar 2005; Gabbard et al. 2009). In both these works, Gabbard and colleagues asked either adults (Gabbard and Ammar 2005; age 19–23) or children (Gabbard et al. 2009) age 7, 9, or 11 to judge whether different visual targets were in reach both when they were in a sitting and a standing position. Crucially, this overestimation was more significant in children than adults and was resistant to postural manipulations. The conflicting findings (underestimation in one, overestimation in the other) from these two studies suggest that structural and functional‐based paradigms rely on different body representations, resulting in different patterns of distortions. However, this hypothesis has never been tested.

To this end, we designed a study in which children aged 6 to 10 were required to estimate their arm length in two tasks. In the first task, we used the widely used body‐landmark paradigm to obtain a structural representation of the forearm and hand in space. In the second task, the same children completed a functional task where they had to judge whether their arm was long enough to touch an object at various distances. This design enabled us to assess how structural and functional representation of the upper limb and their relationship change across age stages in childhood. We hypothesized, based on previous research, that children would underestimate arm length in the structural task but overestimate their reaching abilities in the functional task. Moreover, our second hypothesis was that if structural and functional tasks require two independent representations of the arm, there should be no relationship between these two estimates.

## 2 Methods

### 2.1 Participants

Eighty‐four children (age range 6–10 years old; 37 females, mean age 8.13 yo, SD 1.46) were recruited for the study through a local school. Children were divided into five age groups (6 years old: *N*  =  19 (*F* = 12); 7 yo: *N*  =  15 (*F* = 4); 8 yo: *N*  =  15 (*F* = 6); 9 yo: *N*  =  20 (*F* = 8); 10 yo: *N*  =  15 (*F* = 7)). Written informed consent was obtained from the parents before testing. The study was approved by the local ethical committee (Comitato Etico, Asl 3, Genova, protocol name: IIT_COMP_MIS) and conducted according to the revised Helsinki principles Declaration (World Medical Association 2013).

### 2.2 Protocol

Participants completed two tasks, a functional and structural task. The order of the two tasks was counterbalanced across participants.

**FIGURE 1:**
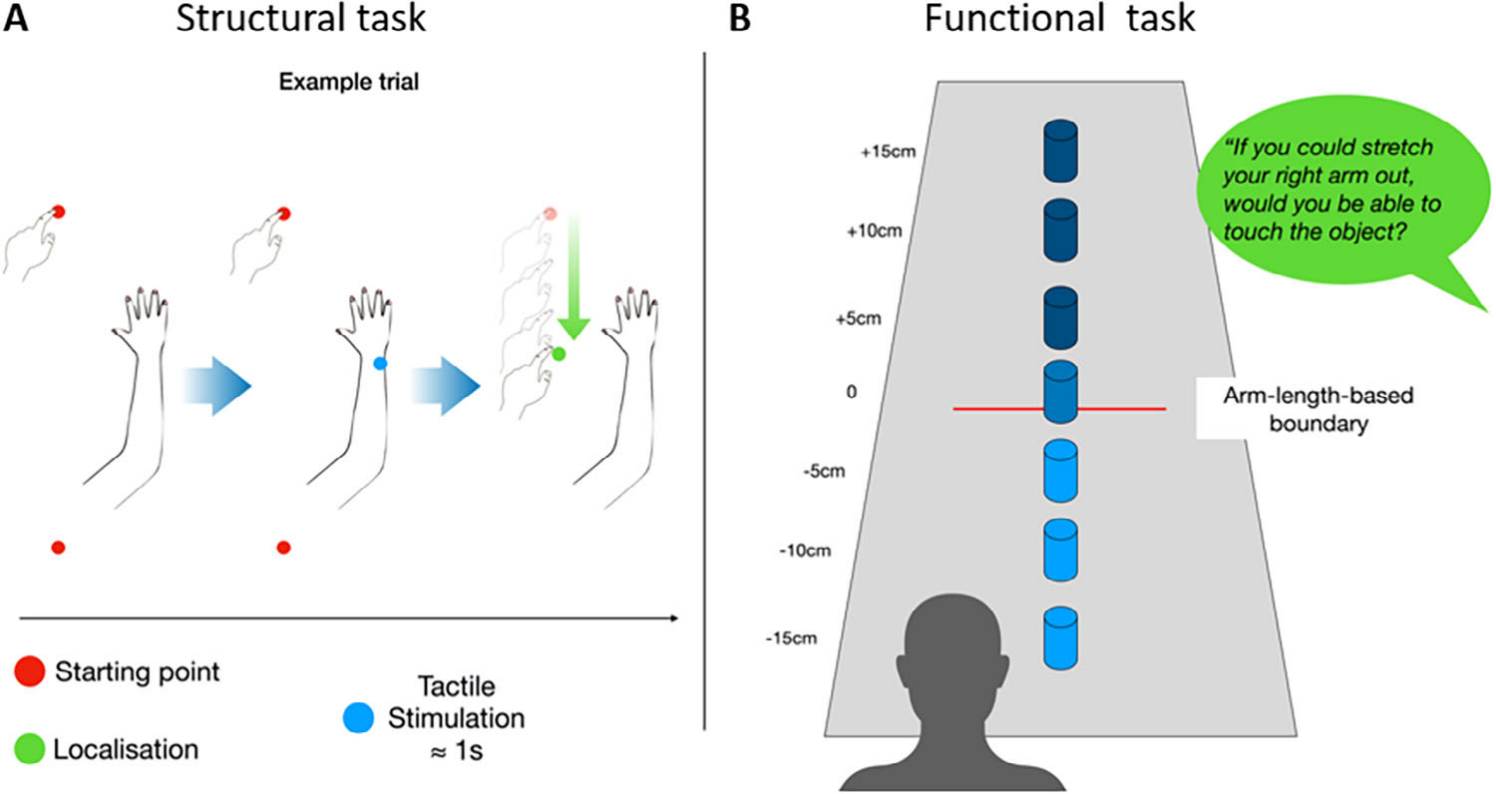
Experimental setup. Graphical illustration of the experimental setup of the two tasks. (A) Structural task. On each trial, participants started with their right arm flat on the table and the left index fingertip resting on one of the two starting points (red dots). The experimenter provided tactile stimulation to one of the three landmarks (blue dot). The child was instructed to slide the left index finger on the table to localize the touched landmark. (B) Functional task. On each trial, an object was placed on the table, and participants were asked to imagine stretching their right arm out and judge whether they could touch said object.

*Structural task*. This task (Figure 1A) was a simplified version of the landmark task used in the literature to assess the estimated length of different body parts (Cardinali et al. 2011). Participants sat blindfolded at a table covered with white paper, with their right arm outstretched and palm down on it. The experimenter touched one of three possible landmarks on the participant's arm: the lateral epicondyle (elbow), the styloid process (wrist), or the tip of the longest finger (in all participants, the middle finger). Participants were asked to report where they felt the touch by sliding the left index finger on the table along a proximal‐distal axis parallel to the right arm without contacting it. At the end of the sliding movement, the experimenter marked the position of their left index finger on the paper. The participant was then instructed to return their same finger to the starting position before the following stimulation (next trial) was delivered. For each child, the starting position was randomized so that the sliding movement direction was proximal to distal in half of the trials and distal to proximal in the other half. Each landmark was stimulated 12 times in a randomized order for 36 trials.

*Functional task*. This task (Figure 1B) required participants to sit in a fixed position against the back of their chair (no moving) and to report whether their arm was long enough to touch an object (a cylinder) placed on the table. Upon arrival and after sitting at a table, participants were asked to close their eyes and stretch their right arm out in front of them while keeping their back in contact with the chair and not leaning forward. This was done for two reasons: the first one was to mark, on the external edge of the table (i.e., in a location not visible to the child), the position of the tip of the index finger to use as a reference for positioning the object on the table (see below). The second one was to give a “freshly performed” movement to imagine. Indeed, at the beginning of each trial, the child was asked: “If you were to stretch your arm out in front of you without leaning forward, as you did before, would you touch the object?” The object was placed at seven different locations along a proximal‐distal line originating on the child's right shoulder. One location (0) was at the point marked by the experimenter as the tip of the longest finger. The other six locations were 5, 10, or 15 cm from location zero, either more proximal (−5, −10, or −15) or more distal (+5, +10, or +15). The object was presented 12 times at each location for 84 trials. The order of presentation was randomized across participants. Participants’ answers (in the form of a yes/no response) were marked by the experiment and later used to build a psychometric curve.

### 2.3 Quantification and Statistical Analyses

*Structural task*. We computed the estimated hand and forearm length as the distance between the average localization of the fingertip‐wrist landmarks and the wrist‐elbow landmarks, respectively. To assess the significance of differences in estimated and actual hand and forearm lengths across age groups, we used mixed‐measures ANOVAs with age as a between‐subjects factor and segment (forearm, hand) and measure (estimated, actual) as within‐subjects factors.

To understand whether age‐related differences were driven by changes in perceptual estimates or physical growth, we analyzed both estimated and actual segment lengths. Previous research has shown that during development (ages 8–16), physical hand size increases while estimated hand size remains relatively stable (Coelho and Gonzalez 2022). Therefore, it was important to consider both components in our analyses. We used mixed‐measures ANOVAs to assess differences in the total upper limb length (sum of hand and arm segments) and followed up significant effects with Bonferroni‐corrected post‐hoc tests.

To align with standard practices in the structural body representation literature, we also conducted secondary analyses using normalized data. Specifically, we calculated percentage distortion using the formula:

$$\begin{array}{ccc} & & [\left(\mathrm{Estimated}\;\mathrm{segment}\;\mathrm{size}-\mathrm{Actual}\;\mathrm{segment}\;\mathrm{size}\right)/\\ & & \;\mathrm{Actual}\;\mathrm{segment}\;\mathrm{size}]\times \;100 \end{array}$$

These values were analyzed using a series of one‐way ANOVAs to assess whether the normalized estimates showed significant distortions across groups.

If the results from these mixed‐measures ANOVAs indicate a distorted body representation (the estimated is different from the real size), it is possible that the distortions arise from opposite directional localization errors (shifts) between each landmark's actual and estimated positions. For example, underestimation of hand size may result from a shift of the fingertip landmark towards the wrist, or a shift of the wrist landmark towards the finger, or both. To explore directional errors in landmark localization, we computed the signed distance between actual and estimated localization of each landmark (in cm) at the single‐subject, single‐trial level. We assigned signs so that negative errors represent shifts toward the body and positive errors represent shifts away from the body. We used mixed effects models to assess the significance of differences in age and landmark on shifts in localization. We considered age and landmark as fixed effects, gender and sliding movement direction as covariates, and subject (random intercept and random slope of landmark) as random effects. For selection of random and fixed effects, we applied a backward model selection procedure, starting from the model with the most complex structure to arrive at a model that included only the significant predictors (see Table S1). We first selected the random effect structure of the model by keeping the full fixed effect structure and using the BIC (Bayesian Information Criterion). This resulted in a preferred model with random intercepts and slopes (BIC = 15690, *p* < 0.01). The BIC rewards model fit and penalizes model complexity. Once the random effects structure was selected, we defined the fixed effects structure using likelihood ratio tests (LRT) between models, which differed for the presence or absence of a predictor. The final model revealed a main effect of landmark (*F*(1.54, 104.8) = 116.5, *p* < 0.01). Indicating that there was an effect of landmark on the participants’ shift in estimates. All analyses were run with R (R Core Team 2014). Mixed effects models were implemented using the *lme4* package. Comparisons across levels of the selected models were performed using the *lsmeans* package.

*Functional task*. For the functional task, we coded participants’ responses as “yes” and “no” and computed, separately for each age group, the psychometric curve of the probability of responding “yes” as a function of object distance. We then fit this data using a Gaussian function. In our experiment, we calculated each participant's actual arm length (i.e., their physical reach limit) and normalized this value to **0**, establishing it as the reference point or threshold for comparison. The resulting PSE values represent participants’ perceived reach in relation to their actual reach. We then calculated the shift from this threshold (i.e., how far participants thought they could reach relative to their actual arm length). As such, **a PSE value of 0 indicates veridical perception**, and **any statistically significant deviation from 0 indicates a perceptual bias** (and the PSE threshold)—either an over‐ or underestimation of arm length. We used ANOVAs to assess the significance of differences in threshold (i.e., the position at which participants were equally likely to answer yes or no) and the slope of the curve across age groups. We used two‐tailed *t*‐tests to compare the point of subjective equivalence (PSE) for each age group against 0.

## 3 Results

### 3.1 Structural Task

**FIGURE 2:**
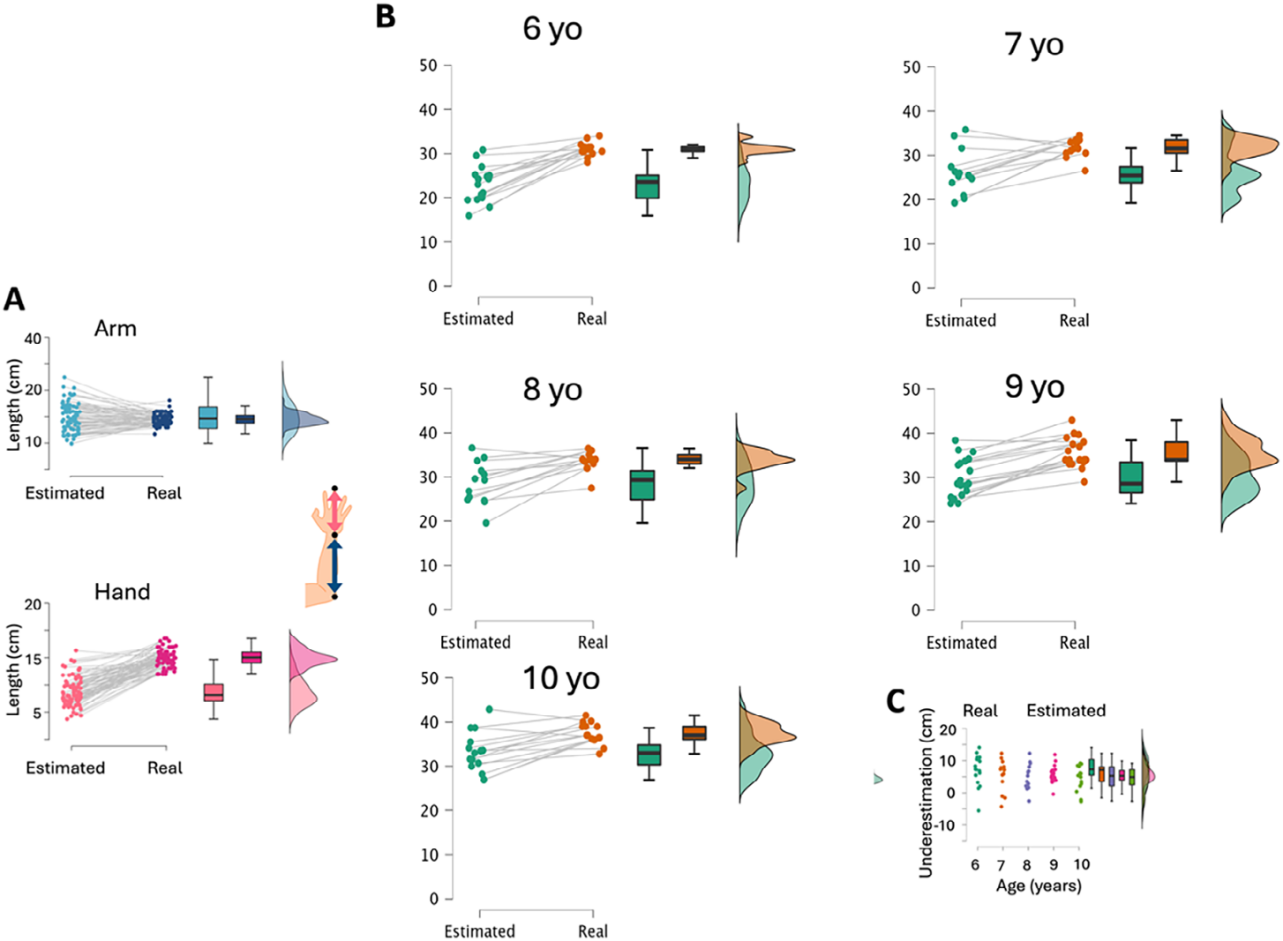
Results of structural task. (A) Results from the Body Landmark task are expressed in cm for each participant and at the group level for the hand (distance wrist–fingertip) and the forearm (distance wrist–elbow). (B) Real and estimated length of the total arm (hand + arm segment) for each age group. Graphs show individual performance (dots and lines) and group performance (bar graph). (C) Amount of underestimation (expressed in cm) of the total arm length for each age group. Dots represent single participants.

A mixed‐measures ANOVA with age as a between‐subjects factor and segment (forearm, hand) and measure (estimated, actual) as within‐subjects factors revealed a significant interaction age*segment (*F*(4, 72) = 4.88, *p* = 0.002, *η* ^2^ = 0.02) and a significant measure*segment interaction (*F*(4, 72) = 92.39, *p* < 0.001, *η* ^2^ = 0.1) (Figure 2A). These effects are further explained by a three‐way interaction age*measure*segment *(F*(4, 72) = 3.54, *p* = 0.011, *η* ^2^ = 0.02). See Table 1 for means and standard errors and statistics and Table 2 to display the mean and absolute errors.

**TABLE 1:** Means and standard errors and statistics of the estimated and actual body part lengths across development.

	6 yo	Statistics	7 yo	Statistics	8 yo	Statistics	9 yo	Statistics	10 yo	Statistics
Estimated forearm	15.44 ± 1.17		17.42 ± 1.42		20.08 ± 1.50		21.03 ± 0.66		24.01 ± 1.23	
Actual forearm	17.62 ± 0.23	*t*(16) = −3.74, *p* = 0.05	17.27 ± 0.46	*t*(13) = −0.154*, p* = 1	19.04 ± 0.422	*t*(13) 1.10, *p* = 1	20.14 ± 0.52	*t*(19) 1.14, *p* = 1	20.83 ± 0.43	*t*(15), 3.61, *p* = 1
Estimated hand	8.28 ± 0.68		8.82 ± 0.52		8.5 ± 0.99		9.00 ± 0.63		9.05 ± 0.59	
Actual hand	13.27 ± 0.22	*t*(16) *=* −*5.60*, *p* < 0.01	14.41 ± 0.20	*t*(13) = −5.91, *p* < 0.01	14.62 ± 0.29	*t*(13) = −6.41, *p* < 0.01	15.58 ± 0.33	*t*(19) = −8.40, *p* < 0.01	16.60 ± 0.27	*t*(15) = −8.52, *p* < 0.01

*Note*: The statistics rows provide the *t*‐test statistics between the estimated and actual segment sizes for each age group.

**TABLE 2:** The mean errors and absolute errors for the structural task.

	Forearm	Hand
Mean error	0.55 ± 0.54	−6.17 ± 0.31
Absolute error	3.64 ± 0.34	6.21 ± 0.30

Post‐hoc comparisons revealed distinct patterns in segment length estimation across age groups. For the hand, the estimated length was consistently smaller than the actual length across all ages. Notably, although the actual length of the hand grew with age, the estimated size remained constant, resulting in greater underestimation as age increased. This replicates previous findings that while physical hand size increases across development, the perceptual size remains consistent (Coelho and Gonzalez 2022). In contrast, for the arm, only the youngest children (6 yo) underestimated its length. All other age groups did not show any significant difference between their actual and estimated forearm size.

As shown in Figure 2B, when considering the total length of the upper limb, these patterns resulted in a main effect of age (*F*(4, 72) = 15.6, *p* < 0.001, *η* ^2^ = 0.07) and a main effect of measure (*F*(1, 72) = 136.42, *p* < 0.001, *η* ^2^ = 0.07). Both actual and estimated total lengths increased with age, as expected. However, the estimated total length was consistently shorter than the actual total length across all age groups (Figure 2).

*Normalized data*: There was a main effect of segment (*F*(1, 72) = 119.66, *p* < 0.01, *η* ^2^ = 0.51) in which children underestimated hand length (−41.27% ± 1.96) compared to forearm length (2.651 ± 2.87), which was relatively accurate. There was a segment by age interaction that approached significance (*F*(4, 72) = 2.16, *p* = 0.08, *η* ^2^ = 0.012). Follow‐up pairwise comparisons revealed that the youngest children (aged 6) underestimated hand and forearm length to a similar extent (*p* = 0.17), whereas the 7–10‐year‐olds all underestimated hand length significantly more (*p*’s > 0.01). Furthermore, our series of one‐sample *t*‐tests showed that children of all ages significantly underestimated hand length (*p*’s < 0.01). With respect to forearm estimation, the 6‐year‐old children marginally underestimated this segment(−12.25 ± 6.90, *p* = 0.095), while the 9‐year‐olds marginally overestimated it (4.63 ± 2.5, *p* = 0.077) and the 10‐year‐olds significantly overestimated forearm length (15.59 ± 5.99, *p* = 0.02).

**FIGURE 3:**
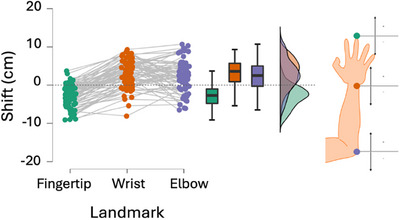
Landmarks localization shift. Distance between actual landmark location and localization expressed in cm for each participant (dots) and at the group level (bar graph). Positive values indicate an overshoot (localization shifted distally) and negative values indicate an undershoot (localization shifted proximally).

*Shift in localization*. The results so far indicate a distortion of hand and arm structural representations across age stages. Mixed effects statistics with age and landmark as fixed factors revealed a significant main effect of landmark, reflecting a proximal shift of the fingertip marker (estimated position: 30.92 ± 0.44, actual position: 33.94 ± 0.39)and a distal shift of the wrist (estimated position: 22.18 ± 0.44, actual position: 19.05 ± 0.25) and elbow markers (estimated position: 2.59 ± 0.48, actual position: 0.13 ± 0.07). See Figure 3 for a visual description. The effect of age did not reach significance (*p* = 0.3).

### 3.2 Functional Task

**FIGURE 4:**
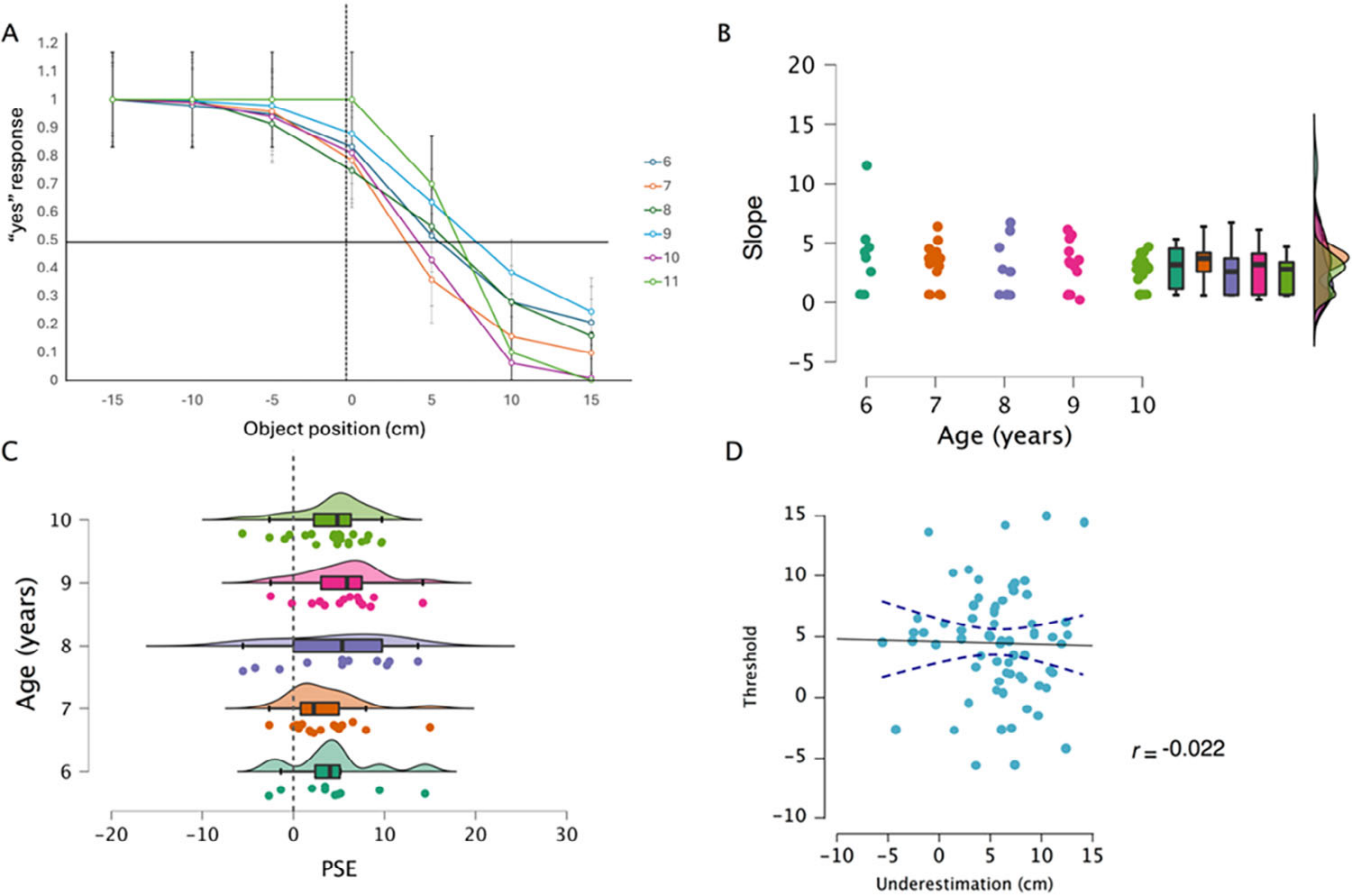
Results of the functional task. (A) The average psychometric curve for each age group, error bars show the standard error of the participants. (B) Slopes value from the psychometric curves drawn for each participant. Dots represent single subject values and boxplots group data. (C) Point of subjective equivalence (PSE) for each age group. Dots represent single‐subject performance and boxplots group data. (D) Correlation plot between threshold data of the reaching judgment task and underestimation data for the whole arm (hand + arm) from the body landmark task.

We first ran a series of two‐tailed *t*‐tests to compare the PSE for each age group against 0 and found that for all groups, PSE values were significantly higher than 0 (all *p*’s < 0.03). This shift forward of the PSE suggests an overestimation of total arm length (Figure 4). We then proceeded to analyze differences among groups. We ran two separate ANOVAs with threshold and slope as dependent variables and age as a factor. We found no significant differences across age groups (threshold: *F*(4, 76) = 0.81, *p* = 0.51, *η* ^2^ = 0.04; slope: *F*(4, 76) = 1.03, *p* = 0.39, *η* ^2^ = 0.05), suggesting a constant overestimation throughout childhood.

### 3.3 Relation Between Tasks

Finally, we looked at whether performance in the two tasks was related. We ran a correlation analysis between the total arm length estimation obtained from the structural task and the threshold value from the reaching judgment (functional) task. We found no significant correlation (Pearson's *r* = 0.02, *p* = 0.58; Figure 4D).

## 4 Discussion

In this study, we aimed to understand how body representations develop in children, whether children overestimate or underestimate the size of their arms and hands, and how this affects their judgment about interacting with an object. To address these questions, we tested a large cohort of children aged 6 to 10 in two tasks: one designed to investigate the structural representation of their upper limb length (landmark task) and another to determine their perceived (functional) reachability of an object by the same limb (reaching judgment task).

Previous studies on arm/hand representation have shown mixed results (depending on task), providing evidence in favor of both overestimation and underestimation (Cardinali et al. 2019; Coelho and Gonzalez 2022; Coelho et al. 2017; Gabbard and Ammar 2005; Gabbard et al. 2009; Mancini et al. 2011; Tamè et al. 2017). Our results show that both under‐ and overestimation of body parts (in this case, the arm) can coexist in children. This finding is in line with our hypothesis that structural and functional body representations are independent representations of the body.

Specifically, we found at the structural level (body landmark task), all ages of children significantly underestimated their hand length but not their forearm length. Follow‐up analyses revealed that underestimation of hand length resulted from fingertip localization being shifted backward and wrist localization being shifted forward (Figure 3). In contrast, elbow localization did not show significant shifts. This shift in landmark localization cannot be attributed to a bias induced by the direction of the sliding movement, as this was randomized across trials (for the same landmark, half of the time, participants reached it from a proximal location and the other half from a distal location, in random order). These results confirm previous results from our group and others (Cardinali et al. 2019; Coelho et al. 2023; Longo et al. 2015; Tamè et al. 2017) indicating a consistent underestimation of hand length, contributing to an overall underestimation of total arm length in both children and adults at the structural level.

A recent study suggests that the distortions at the structural level (landmark task) do not reflect inaccurate body representations but are actually a byproduct of a noisy somatosensory signal encoding (Peviani et al. 2024). Therefore, an alternative explanation for our results is that during development children rely on a noisier somatosensory system, leading to significant underestimation of hand/arm length. This is a topic that should be explored in the future. Moreover, as previous work has shown that children have greater variability in both somatosensory and spatial perception (Cowie et al. 2010), their overestimation in the reaching judgment task could originate from a broader developmental trend of overestimation of spatial representations in action‐based estimations. Further work should explore if this extends to other spatial judgments involving peripersonal and extrapersonal space.

At the functional level, we found a significant overestimation of the maximal reachable point for the reaching judgment task across all age groups. This suggests a constant overestimation of arm length throughout childhood and is in line with the findings by Gabbard et al. (2009). This alignment with Gabbard's findings is particularly intriguing given the differing instructions. In our study, we instructed children to imagine lifting the arm while keeping their back against the chair and then asked whether their arm was long enough to touch the object. The focus was on arm length estimation. In contrast, Gabbard et al. (2009) designed the task to study reaching space, and instructions focused more on the mental simulation of a reaching movement toward the object rather than the effector length. In other words, in Gabbard, the focus was on the mental simulation of the action, while in our experiment, the emphasis was on the arm‐length knowledge that is needed to judge whether an object is reachable or not. Despite this difference, we obtained the same results.

It is possible that when imagining stretching their arm, children in our study also imagined leaning forward, as one would typically do in real life to reach an object. If the leaning movement was considered, it would be fascinating to learn that a limb representation used to solve a motor‐related task includes features of such movement. In other words, when the knowledge of the arm length is needed for action performance, it is provided in a format that contains morphological and movement‐related features. This is an exciting topic for future studies. If this is the case, it would suggest that one representation of arm length guides both perception and action. This would mean that the underestimation of arm length found in the structural task is being considered during the functional task, causing the shifted PSEs. In other words, the functional task measures the bias of action based on the distorted structural representation. This speculation is reminiscent of work on number line estimation, which has shown a developmental trend (Cohen and Sarnecka 2014; Siegler and Opfer 2003; Siegler et al. 2009). In these studies, participants are asked to estimate the location of a number on a line. In one such study, children were found to use different strategies compared to adults, suggesting that the developmental trend in this spatial ability is reflecting task‐specific measurements and not alterations in the comprehension of numeric quantities across development (Cohen and Sarnecka 2014). It is possible that a similar phenomenon is occurring in the current study. If this is the case, it would suggest that task format results in the different distortions of body representation.

Despite this intriguing possibility, we believe, however, that our results align more closely with the proposal of multiple independent body representations (de Haan and Dijkerman 2020; De Vignemont 2007, 2010; Longo 2015), as we found no correlation between the estimated total arm length derived from the structural and functional tasks. If the same body representation was mediating both tasks, we would expect to see a relationship (even a negative one) between the two tasks. This lack of correlation supports our second hypothesis that multiple body representations exist not only in adults but also in children, and their access is determined by the task to be performed and the information necessary for that to happen (De Vignemont 2007, 2010; Longo 2015; Tamè et al. 2017). In the work of Tamè and colleagues (2017), they found unique distortions in three different body representation tasks and that these distortions persisted despite different instructions/prior beliefs. Our results replicate part of these findings, mainly that the same group of participants exhibit unique distortions in tasks that measure different body representations. The debate about the number of body representations stored and updated by humans is ongoing (Kammers et al. 2010; De Vignemont 2007; Longo et al. 2010; Medina 2022). Further imaging research is critical to understand if these tasks measure independent body representations or if they are in fact task format results.

Finally, our finding that overestimation and underestimation of arm and hand length coexist in the same group of children aligns with broader trends in spatial cognition, where perceptual distortions are common. Previous research has shown that people tend to compress distances within peripersonal space but expand distances in extrapersonal space, a pattern thought to reflect task demands and action affordances (Loomis et al. 1992; Coello and Delevoye‐Turrell 2007). The overestimation of reachability we observed in children may reflect an action‐specific distortion, where perceived space expands to accommodate potential movements, consistent with the action capabilities hypothesis (Proffitt et al. 2003).

In conclusion, we found that children both overestimate and underestimate the total length of their arms and hands based on the specific demands of a task, with no observable correlation between these estimates. We argue that these results support the coexistence of multiple, independent body representations in children. Future studies should aim to understand whether and how these representations interact to support accurate motor control and perceptual processing across the lifespan.

## Author Contributions

**Lucilla Cardinali**: conceptualization, investigation, writing – original draft, methodology, validation, data curation, formal analysis. **Cristina Becchio**: conceptualization, funding acquisition, writing – review and editing, supervision. **Lara Coelho**: writing – review and editing, formal analysis, writing – original draft. **Monica Gori**: conceptualization, funding acquisition, writing – review and editing, supervision, resources.

## Conflicts of Interest

The authors declare no conflicts of interest.

## Supporting information



Supporting Information

## Data Availability

Upon acceptance all data will be uploaded to the Zonodo repository.

## References

[desc70035-bib-0001] Anema, H. A. , M. J. van Zandvoort , E. H. de Haan , et al. 2009. “A Double Dissociation Between Somatosensory Processing for Perception and Action.” Neuropsychologia 47, no. 6: 1615–1620. 19038277 10.1016/j.neuropsychologia.2008.11.001

[desc70035-bib-0002] Bassolino, M. , and C. Becchio . 2022. “The ‘Hand Paradox’ Distorted Representations Guide Optimal Actions.” Trends in Cognitive Sciences 27, no. 1: 7–8. 36418208 10.1016/j.tics.2022.09.010

[desc70035-bib-0003] Bassolino, M. , A. Finisguerra , E. Canzoneri , A. Serino , and T. Pozzo . 2015. “Dissociating Effect of Upper Limb Non‐Use and Overuse on Space and Body Representations.” Neuropsychologia 70: 385–392. 25462198 10.1016/j.neuropsychologia.2014.11.028

[desc70035-bib-0004] Caggiano, P. , L. Veronelli , L. Mora , L. S. Arduino , M. Corbo , and G. Cocchini . 2020. “The Downsized Hand in Personal Neglect.” Journal of Clinical and Experimental Neuropsychology 42, no. 10: 1072–1084. 33203298 10.1080/13803395.2020.1843603

[desc70035-bib-0005] Campos, J. J. , D. I. Anderson , M. A. Barbu‐Roth , E. M. Hubbard , M. J. Hertenstein , and D. Witherington . 2000. “Travel Broadens the Mind.” Infancy 1, no. 2: 149–219. 32680291 10.1207/S15327078IN0102_1

[desc70035-bib-0006] Cardinali, L. , C. Brozzoli , C. Urquizar , R. Salemme , A. Roy , and A. Farnè . 2011. “When Action Is Not Enough: Tool‐Use Reveals Tactile‐Dependent Access to Body Schema.” Neuropsychologia 49, no. 13: 3750–3757. 21971306 10.1016/j.neuropsychologia.2011.09.033

[desc70035-bib-0007] Cardinali, L. , A. Serino , and M. Gori . 2019. “Hand Size Underestimation Grows During Childhood.” Scientific Reports 9, no. 1: 13191. 31520003 10.1038/s41598-019-49500-7 PMC6744419

[desc70035-bib-0008] Coelho, L. A. , and C. L. Gonzalez . 2022. “Growing Into Your Hand: The Developmental Trajectory of the Body Model.” Experimental Brain Research 1–11. 10.1007/s00221-021-06241-2 34654947

[desc70035-bib-0009] Coelho, L. A. , and C. L. Gonzalez . 2024. “Perception, Action, and the Body Model.” Neuropsychologia 196: 108853. 38490535 10.1016/j.neuropsychologia.2024.108853

[desc70035-bib-0010] Coelho, L. A. , R. Lee , and C. L. Gonzalez . 2023. “The Distorted Hand: Systematic but ‘Independent’ Distortions in Both Explicit and Implicit Hand Representations in Young Female Adults.” Experimental Brain Research 241, no. 1: 175–186. 36414752 10.1007/s00221-022-06511-7

[desc70035-bib-0011] Coelho, L. A. , G. Zaninelli , and C. L. Gonzalez . 2017. “A Kinematic Examination of Hand Perception.” Psychological Research 81: 1224–1231. 27738751 10.1007/s00426-016-0815-9

[desc70035-bib-0012] Coello, Y. , and Y. Delevoye‐Turrell . 2007. “Embodiment, Spatial Categorisation and Action.” Consciousness and Cognition 16, no. 3: 667–683. 17728152 10.1016/j.concog.2007.07.003

[desc70035-bib-0013] Cohen, D. J. , and B. W. Sarnecka . 2014. “Children's Number‐Line Estimation Shows Development of Measurement Skills (Not Number Representations).” Developmental Psychology 50, no. 6: 1640. 24512172 10.1037/a0035901 PMC5087800

[desc70035-bib-0014] Cowie, D. , J. M. Gottwald , L.‐A. Bird , and A. J. Bremner . 2022. “The Role of Hand Size in Body Representation: A Developmental Investigation.” Scientific Reports 12, no. 1: 19281. 36369342 10.1038/s41598-022-23716-6 PMC9652309

[desc70035-bib-0015] Cowie, D. , J. Atkinson , and O. Braddick . 2010. “Visual and Haptic Judgments of Object Size in Children: Effects of Age and Stimulus Modality.” Developmental Science 13, no. 1: 151–161.20121871

[desc70035-bib-0016] de Haan, E. H. F. , and H. C. Dijkerman . 2020. “Somatosensation in the Brain: A Theoretical Re‐Evaluation and a New Model.” Trends in Cognitive Sciences 24, no. 7: 529–541. 10.1016/j.tics.2020.04.003. 32430229

[desc70035-bib-0017] De Vignemont, F. 2007. “How Many Representations of the Body?” Behavioral and Brain Sciences 30, no. 2: 204–205.

[desc70035-bib-0018] De Vignemont, F. 2010. “Body Schema and Body Image—Pros and Cons.” Neuropsychologia 48, no. 3: 669–680. 19786038 10.1016/j.neuropsychologia.2009.09.022

[desc70035-bib-0019] Dijkerman, C. , and B. Lenggenhager . 2018. “The Body and Cognition: The Relation Between Body Representations and Higher Level Cognitive and Social Processes.” Cortex: A Journal Devoted to the Study of the Nervous System and Behavior 104: 133–139. 29914632 10.1016/j.cortex.2018.06.001

[desc70035-bib-0020] Gabbard, C. , and D. Ammar . 2005. “Visual Cues and Perceived Reachability.” Brain and Cognition 59, no. 3: 287–291. 16154676 10.1016/j.bandc.2005.07.006

[desc70035-bib-0021] Gabbard, C. , A. Cordova , and S. Lee . 2009. “Do Children Perceive Postural Constraints When Estimating Reach or Action Planning?” Journal of Motor Behavior 41, no. 2: 100–105. 19201680 10.3200/JMBR.41.2.100-105

[desc70035-bib-0022] Galigani, M. , N. Castellani , B. Donno , et al. 2020. “Effect of Tool‐Use Observation on Metric Body Representation and Peripersonal Space.” Neuropsychologia 148: 107622. 32905815 10.1016/j.neuropsychologia.2020.107622

[desc70035-bib-0023] Gallagher, S. , and J. Cole . 1995. “Body Image and Body Schema in a Deafferented Subject.” Journal of Mind and Behavior 16, no. 4: 369–389.

[desc70035-bib-0024] Head, H. , and G. Holmes . 1911. “Sensory Disturbances From Cerebral Lesions.” Brain 34, no. 2–3: 102–254.

[desc70035-bib-0025] Kammers, M. P. , J. Mulder , F. De Vignemont , and H. C. Dijkerman . 2010. “The Weight of Representing the Body: Addressing the Potentially Indefinite Number of Body Representations in Healthy Individuals.” Experimental Brain Research 204: 333–342. 19771419 10.1007/s00221-009-2009-9 PMC2895870

[desc70035-bib-0026] Krajenbrink, H. , J. Lust , P. Wilson , and B. Steenbergen . 2020. “Development of Motor Planning in Children: Disentangling Elements of the Planning Process.” Journal of Experimental Child Psychology 199: 104945. 32750601 10.1016/j.jecp.2020.104945

[desc70035-bib-0027] Linkenauger, S. A. , J. K. Witt , J. Z. Bakdash , J. K. Stefanucci , and D. R. Proffitt . 2009. “Asymmetrical Body Perception: A Possible Role for Neural Body Representations.” Psychological Science 20, no. 11: 1373–1380. 19788528 10.1111/j.1467-9280.2009.02447.x PMC2858772

[desc70035-bib-0028] Longo, M. R. 2015. “Types of Body Representation.” In Perceptual and Emotional Embodiment, edited by Y. Coello and M. H. Fischer , 125–142. Routledge.

[desc70035-bib-0029] Longo, M. R. 2022. “Distortion of Mental Body Representations.” Trends in Cognitive Sciences 26, no. 3: 241–254. 34952785 10.1016/j.tics.2021.11.005

[desc70035-bib-0030] Longo, M. R. , and P. Haggard . 2010. “An Implicit Body Representation Underlying Human Position Sense.” Proceedings of the National Academy of Sciences 107, no. 26: 11727–11732. 10.1073/pnas.1003483107 PMC2900654 20547858

[desc70035-bib-0031] Longo, M. R. , E. Azañón , and P. Haggard . 2010. “More Than Skin Deep: Body Representation Beyond Primary Somatosensory Cortex.” Neuropsychologia 48, no. 3: 655–668. 19720070 10.1016/j.neuropsychologia.2009.08.022

[desc70035-bib-0032] Longo, M. R. , and P. Haggard . 2012a. “A 2.5‐D Representation of the Human Hand.” Journal of Experimental Psychology: Human Perception and Performance 38, no. 1: 9. 21895388 10.1037/a0025428

[desc70035-bib-0033] Longo, M. R. , and P. Haggard . 2012b. “Implicit Body Representations and the Conscious Body Image.” Acta Psychologica 141, no. 2: 164–168. 22964057 10.1016/j.actpsy.2012.07.015

[desc70035-bib-0034] Longo, M. R. , S. Mattioni , and N. Ganea . 2015. “Perceptual and Conceptual Distortions of Implicit Hand Maps.” Frontiers in Human Neuroscience 9: 656. 26733842 10.3389/fnhum.2015.00656 PMC4679851

[desc70035-bib-0035] Loomis, J. M. , J. A. Da Silva , N. Fujita , and S. S. Fukusima . 1992. “Visual Space Perception and Visually Directed Action.” Journal of Experimental Psychology: Human Perception and Performance 18, no. 4: 906. 1431754 10.1037//0096-1523.18.4.906

[desc70035-bib-0036] Mancini, F. , M. R. Longo , G. D. Iannetti , and P. Haggard . 2011. “A Supramodal Representation of the Body Surface.” Neuropsychologia 49, no. 5: 1194–1201. 21199662 10.1016/j.neuropsychologia.2010.12.040

[desc70035-bib-0037] Medina, J. 2022. “Distinguishing Body Representations.” In The Routledge Handbook of Bodily Awareness, edited by A. J. T. Alsmith and M. R. Longo , 150–160. Routledge.

[desc70035-bib-0038] Paillard, J. 1999. “Body Schema and Body Image‐a Double Dissociation.” Motor Control, Today and Tomorrow 197: 214.

[desc70035-bib-0039] Peviani, V. C. , L. E. Miller , and W. P. Medendorp . 2024. “Biases in Hand Perception Are Driven by Somatosensory Computations, Not a Distorted Hand Model.” Current Biology 34, no. 10: 2238–2246. 38718799 10.1016/j.cub.2024.04.010

[desc70035-bib-0040] Proffitt, D. R. , J. Stefanucci , T. Banton , and W. Epstein . 2003. “The Role of Effort in Perceiving Distance.” Psychological Science 14, no. 2: 106–112. 12661670 10.1111/1467-9280.t01-1-01427

[desc70035-bib-0041] Shi, P. , and X. Feng . 2022. “Motor Skills and Cognitive Benefits in Children and Adolescents: Relationship, Mechanism and Perspectives.” Frontiers in Psychology 13: 1017825. 36478944 10.3389/fpsyg.2022.1017825 PMC9721199

[desc70035-bib-0042] Siegler, R. S. , and J. E. Opfer . 2003. “The Development of Numerical Estimation: Evidence for Multiple Representations of Numerical Quantity.” Psychological Science 14, no. 3: 237–250. 12741747 10.1111/1467-9280.02438

[desc70035-bib-0043] Siegler, R. S. , C. A. Thompson , and J. E. Opfer . 2009. “The Logarithmic‐to‐Linear Shift: One Learning Sequence, Many Tasks, Many Time Scales.” Mind, Brain, and Education 3, no. 3: 143–150.

[desc70035-bib-0044] Tamè, L. , N. Bumpus , S. A. Linkenauger , and M. R. Longo . 2017. “Distorted Body Representations Are Robust to Differences in Experimental Instructions.” Attention, Perception, & Psychophysics 79: 1204–1216. 10.3758/s13414-017-1301-1 28205054

[desc70035-bib-0047] Team, R. C. 2014. R: A Language and Environment for Statistical Computing. http://www.R-project.org.

[desc70035-bib-0045] Von Hofsten, C. 2004. “An Action Perspective on Motor Development.” Trends in Cognitive Sciences 8, no. 6: 266–272. 15165552 10.1016/j.tics.2004.04.002

[desc70035-bib-0046] World Medical Association . 2013. “World Medical Association Declaration of Helsinki: Ethical Principles for Medical Research Involving Human Subjects.” Jama 310, no. 20: 2191–2194. 24141714 10.1001/jama.2013.281053

