# Supplementary material for: Underestimation and Overestimation of Hand and Arm Length Coexist in Children
Source: Dev Sci. 2025 Jun 3;28(4):e70035. doi: 10.1111/desc.70035 (PMC12131706; doi:10.1111/desc.70035)
Supplement: Supplementary file 1 — Supporting Information [file DESC-28-e70035-s001.docx]

Supplementary Table 1: Summary of model selection procedure for random and fixed effects. A backward model selection approach was used, starting from the most complex model. Random effects were selected using BIC, resulting in a model with random intercepts and slopes (BIC = 15690, p < .01). Fixed effects were selected using likelihood ratio tests (LRT) comparing models with and without individual predictors.

| SHIFT | | | | | |
| --- | --- | --- | --- | --- | --- |
| **Random effects structure** | | | | | |
| Model | Random effects | Df | BIC | Deviance | p (LRT) |
| **M1** | **Participant (intercept and Landmark slope)** | **22** | **15690** | **15674** | **<.01** |
| M2 | Participant (intercept) | 17 | 17638 | 17615 | NA |
|  |  |  |  |  |  |
| **Fixed effects structure** | | | | | |
| M3 | Age^x^Landmark, Gender, Direction | 62 | 15684 | 15492 | NA |
| M4 | Age^x^Landmark, Gender | 32 | 15681 | 15498 | .17 |
| M5 | Age^x^Landmark, Direction | 32 | 15676 | 15492 | .3 |
| M6 | Age^x^Landmark | 17 | 15674 | 15498 | **.35** |
| M7 | Age + Landmark | 9 | 15625 | 15513 | **.42** |
| M8 | Age | 7 | 15759 | 15663 | **.3** |
| **M9** | **Landmark** | **5** | **15604** | **15524** | **<.01** |
